# Friendships that money *can* buy: financial security protects health in retirement by enabling social connectedness

**DOI:** 10.1186/s12877-019-1281-1

**Published:** 2019-11-21

**Authors:** Tegan Cruwys, Catherine Haslam, Niklas K. Steffens, S. Alexander Haslam, Polly Fong, Ben C. P. Lam

**Affiliations:** 1 Research School of Psychology, The Australian National University, Canberra, ACT 2601 Australia; 2 School of Psychology, University of Queensland, Brisbane, Australia

**Keywords:** Loneliness, Social capital, Belonging, Healthy ageing, Social determinants, Mental health, Retirement

## Abstract

**Background:**

Research on the health and wellbeing of retirees has tended to focus on financial security and financial planning. However, we suggest that one reason why financial security is important for retirees is that it enables social connectedness, which is critical for healthy ageing.

**Methods:**

This paper tests this hypothesis cross-sectionally (*N* = 3109) and longitudinally (*N* = 404) using a population-weighted mixed effects mediation model in two nationally representative samples of Australian retirees.

**Results:**

Analyses provide robust support for our model. Subjective financial security predicted retiree health cross-sectionally and longitudinally. Social connectedness also consistently predicted mental health and physical health, on average four times more strongly than financial security. Furthermore, social connectedness partially accounted for the protective effect of subjective financial security.

**Conclusions:**

We discuss the implications of these findings for public health, with a particular emphasis on how social connectedness can be better supported for people transitioning to retirement.

As people approach retirement, they typically become increasingly aware of, and feel pressure to engage in, financial planning. Inadequate or uncertain finances have been cited as primary reasons for people delaying retirement [1] and are a leading cause of distress and poor health among retirees [2, 3]. There is evidence, too, that financial status is not only a robust predictor of health and life expectancy in the general population [4], but also becomes an even stronger predictor of health as people age [5]. Indeed, a substantial industry has evolved to address this concern, with superannuation, annuity, and pension schemes providing a raft of services that promise to provide financial planning and management as people approach retirement.

Some researchers have found that decades of favourable policy and investment have meant that a growing proportion of retirees now have ample assets, with many Baby Boomer retirees in advanced economies enjoying a more comfortable standard of living than they did during their working lives [6, 7]. In spite of this, it continues to be the case that a large minority of people leaving the workforce struggle to adjust to retirement and experience a decline in health [8]. It is also the case that financial planning services are more likely to be accessed in the lead-up to retirement by those who need them least (i.e., by people who are wealthier and more financially savvy [9]). This has led to increasing criticism of the emphasis placed on financial planning. For instance, researchers have suggested that the emphasis on finances is disproportionate and that other determinants of retiree health have been relatively neglected: notably, the availability of health services, community engagement, and the circumstances leading to retirement [10, 11].

In the present paper, we propose that a key, and often overlooked, reason why financial security matters in retirement is because finances provide an important means to increase and maintain one’s *social connectedness*. The link between financial security and social connectedness has received little research attention, and what is known comes from outside the retirement context. This work suggests that people living in poverty typically have less social capital [12], are more likely to experience loneliness [13], and tend to spend less time with friends [14]. For instance, in two studies of first-year university students, people from higher socioeconomic status (SES) backgrounds were found to have more social group memberships [15, 16]. The argument underpinning this research, put simply, is that *capital begets capital*. In other words, financial resources increase the availability of other resources, including social connectedness.

Social connectedness, in turn, is a robust and causal predictor of health, both in retirees and in the population more generally [17–21]. For instance, one study found that people who were able to maintain their social connections into retirement not only experienced improved wellbeing but also had a reduced risk of mortality [22]. Evidence suggests that there are likely to be multiple mechanisms that underpin this effect, including the capacity for social connectedness to provide people with a sense of meaning and purpose in life [23] and to provide a basis for the provision and receipt of social support [24].

What we do not yet know is whether, for retirees, social connectedness is one of the resources that is made available through access to financial capital – and whether this, in turn, can partially explain why financial security is important for retiree health. This is because few studies have examined the relationship between the three constructs of financial security, social connectedness, and health together. However, we might predict that this indirect relationship exists, given a broader body of work suggesting that socioeconomic disadvantage is associated with fewer social ties. In particular, two lines of work provide clues to this relationship. The first comes from evidence that having more money does not, in and of itself, make people happier – instead, it is only when money is spent on experiences, such as social activities, that wealth is associated with greater wellbeing [25]. The second comes from evidence that, although financial planning does predict adjustment among retirees, after including social variables in the analysis (e.g., opportunities to acquire new group memberships) this relationship is no longer significant [26]. This pattern is consistent with our hypothesis - that financial security is beneficial for retirees’ health and wellbeing because it enables them to stay socially connected (not yet been tested directly).

Here, we evaluate the relationship between financial security, social connectedness, and health in a sample of retirees drawn from population data. In line with the foregoing logic, our three predictions are as follows:

- Hypothesis 1 (H1): Retirees’ financial security will predict their health.
- Hypothesis 2 (H2): Retirees’ social connectedness will predict their health, after controlling for financial security.
- Hypothesis 3 (H3): There will be an indirect effect of financial security on retirees’ health via social connectedness.

## Method

### Participants and design

Respondents were drawn from a nationally representative population sample of Australian residents in the Housing, Income and Labour Dynamics in Australia survey (HILDA, [27]). HILDA uses a stratified three-stage clustered design and samples all members of selected households on an annual basis. This dataset is recognised internationally as having some of the strongest survey methodology due to its high retention, systematic sampling strategy, and high data quality [28].

Two samples were taken from the most recent waves of HILDA data available (Waves 14 and 15, collected in 2014 and 2015; analysed in 2019). The first (*N* = 3109) was a cross-sectional sample of retirees from Wave 15 who described their employment status as “completely retired” and who did not have missing data on any of the measures of interest. The second (*N* = 404) was a longitudinal sample of workers transitioning to retirement between Waves 14 and 15. These respondents indicated at Wave 15 that they had transitioned to retirement in the last year, and/or listed themselves as employed in Wave 14 and ‘completely retired’ in Wave 15. The demographic characteristics of each sample are reported in Table 1. We excluded participants who were younger than 45 years old on the basis that they were likely to re-enter the workforce (consistent with recommendations of the Australian Bureau of Statistics [29]).

**Table 1:** Sample Demographics

	Cross-sectional sample of retirees (*N* = 3109) ^a^		Longitudinal sample transitioning to retirement (*N* = 404) ^b^	
Gender	56.0% female		50.5% female	
Age	*M* = 70.70 (*SD* = 9.68),		*M* = 65.74 (*SD* = 8.35)	
	Range 45–98		Range 45–94	
Education	Less than Year 12	46.9%	Less than Year 12	33.9%
	Year 12	8.4%	Year 12	7.4%
	Certificate or Diploma	30.6%	Certificate or Diploma	38.1%
	University	14.0%	University	20.3%
Subjective financial security	Very poor	0.9%	Very poor	2.2%
	Poor	3.2%	Poor	3.2%
	Just getting along	24.3%	Just getting along	27.2%
	Reasonably comfortable	56.6%	Reasonably comfortable	53.7%
	Very comfortable	13.8%	Very comfortable	12.6%
	Prosperous	1.2%	Prosperous	1.0%
Household income band	*M* = 6.47 (*SD* = 2.55)		*M* = 6.93 (*SD* = 2.82)	
	Range: 1–13		Range: 1–13	
Social connectedness	*M* = 5.36 (*SD* = 1.08)		*M* = 5.39 (*SD* = 1.07)	
	Range: 1–7		Range: 2.20–7	
Mental health	*M* = 73.83 (*SD* = 18.52)		*M* = 75.02 (*SD* = 17.20)	
	Range: 0–100		Range: 8–100	
Physical health	Poor	9.8%	Poor	7.7%
	Fair	28.4%	Fair	26.0%
	Good	38.5%	Good	34.7%
	Very good	19.7%	Very good	25.2%
	Excellent	3.6%	Excellent	4.7%

^a^Cross-sectional sample is weighted to increase its representativeness of the Australian population. Approximately 11% of the cross-sectional sample was also included in the longitudinal sample. Cross-sectional analyses were repeated with these respondents excluded and the results were not affected. Therefore, the overlapping respondents were retained in order to preserve population-representativeness.

^b^Descriptive statistics for the longitudinal sample are reported for respondents at the post-retirement transition time point.

Testing predictions in both samples allows us to establish (a) the generalizability of our hypotheses to a large representative sample of retirees, and (b) the direction of relationships by modelling the effect of *change* in these constructs during the retirement transition (assessing whether they persist when controlling for baseline financial security, social connectedness, and health status prior to retirement).

### Measures

#### Social connectedness

Social connectedness has been conceptualised and measured in diverse ways; ranging from objective network size, friendship quality, to civic participation. Increasingly though, evidence shows that this construct is best captured subjectively [30]. For example, a meta-analysis of over 300,000 people found that subjective, complex indicators of social connectedness outperformed objective indicators in predicting mortality [31]. There is also theoretical justification for the use of subjective indicators. In particular, the social identity approach argues that social relationships are only likely to influence our health when underpinned by strong psychological connection, as reflected in a sense of belonging, affiliation, and identification [32]. For these reasons we focused on a subjective measure of social connectedness that assessed respondents’ sense of belonging, connection, and support received from others. This 10-item scale includes items such as “When I need someone to help me out, I can usually find someone” and “I often feel very lonely” (reverse scored) measured on a 7-point scale from 1 “strongly disagree” to 7 “strongly agree”. This scale has been used extensively in prior research and has a reliability of .84 across HILDA waves [17, 33].

#### Financial security

Financial security was operationalized in terms of both (1) subjective financial security, and (2) objective income.

The subjective rating of financial security came from the International Survey of Economic Attitudes [34] comprising a single item asking respondents to rate their “prosperity given current needs and financial responsibilities” on a six-point scale from 1 “prosperous” to 6 “very poor”. This scale was reversed for the purposes of our analysis so that higher scores indicated greater financial security.

The objective measure of financial security was gross household income, classified in one of 13 bands ranging from (1) “negative or 0 income” to (13) “AUD200,000+”. All sources of income — from investments, pensions, and other employed members of the household — were included [35]. The median income band in our samples in 2015 was 6, corresponding to a 2015 household income of AUD40,000–49,999. As one would expect for a sample of retirees, this is below the national median of gross household income of AUD84,032 for 2015–2016 [36]. Also as expected, the longitudinal sample experienced a median decline in their income from pre-retirement (band 7) to post-retirement (band 6).

#### Health

Two indices capturing (1) mental health and (2) physical health were used. Mental health was assessed using the five-item Mental Health Inventory from the well-validated MOS SF-36 scale [37]. Respondents are asked how often, in the past four weeks, they experienced symptoms of depression and anxiety (e.g., “felt down”), measured on 6-point scales from 1 “all of the time” to 6 “none of the time”. As recommended by the scale’s authors, ratings were transformed into a score from 0 to 100, where higher scores indicated better mental health.

Physical health was assessed using a one-item self-assessed global health indicator, which is widely used and found to be a reliable and valid predictor of chronic disease and longevity [38]. Respondents were asked “In general, would you say your health is …” and responded on a five-point scale from 1 “Excellent” to 5 “Poor”. This scale was reversed for the purposes of our analysis, so that higher scores were indicative of better physical health.

#### Covariates

Respondents’ age, gender, and education were included as covariates in the analysis to rule out the possibility that the findings were attributable to systematic differences on these variables. Age was measured as a continuous variable at Wave 15, gender was categorically coded as 1 “male”, 2 “female”, or as missing, and education had nine ordinal levels ranging from 1 “Year 11 or below” to 9 “Postgraduate – masters or doctorate”.

### Analytic approach

A multi-level modelling framework was used to assess our predictions. Each model included random intercepts for household (at level 2) and postcode (at level 3) clustering in both the correlational and longitudinal analyses, to account for the nested structure of the dataset. The cross-sectional sample was population weighted to maintain representativeness using weights provided by the Melbourne Institute [39]. All numeric variables were scaled to provide beta coefficients in the output. These analyses of existing data were approved by the ethics committee at the researchers’ university (#2017001606).

To test H1 and H2, a sequence of four linear mixed models were conducted (using R package *lme4*, [40]). Model 1 was the null model. Model 2 added covariates and, in the longitudinal analyses, measures of the three focal variables at baseline (pre-retirement) were included. Model 3 added financial security to test H1. Model 4 added social connectedness to test H2. To test H3, a mixed effects mediation model (using R package *mediation*, [41]) with random intercepts for household was conducted with 1000 bootstrapped samples to determine whether the protective effects of financial security on health were attributable, in part, to their indirect effect via social connectedness. Due to space constraints, the primary analyses reported in text include covariates (age, gender, and education) and focus on the operationalisations of subjective financial security (rather than household income). However, the analyses were repeated using other combinations and are reported in the Additional file 1 (as described below, see Robustness Checks).

## Results

### Cross-sectional analyses

#### Mental health

Model 1 included random intercepts for household (accounting for 39% of the variance) and postcode (accounting for 5% of the variance). Model 2 included the covariates of age, sex, and education. Each was a significant predictor indicating that people who were male, older, or better educated were likely to experience better mental health. Confirming H1, Model 3 showed that subjective financial security significantly predicted mental health, β = .29, *p* < .001; Confidence Interval (CI): .25, .33. Confirming H2, Model 4 revealed that social connectedness also significantly predicted mental health, β = .44, *p* < .001; CI: .41, .47. Log-likelihood ratio tests between each subsequent model (1–4) were all significant χ^2^(1–4) = 78.95–654.29, *p*s < .001, indicating that model fit was improved with each additional predictor. Full details of the cross-sectional mixed effects models are provided in Table 2.

**Table 2:** Cross-sectional linear mixed models assessing H1 and H2

	Cross-sectional sample of retirees (*N* = 3109)					
	DV: Mental health			DV: Physical health		
Model 1 – Random intercepts for household and postcode						
Model 2 - Covariates	β	*SE*	*p*	β	*SE*	*p*
Gender	.01	.03	.001	−.11	.03	.001
Age	.01	.002	<.001	−.001	.002	.660
Education	.03	.006	<.001	.04	.007	<.001
Model 3 – H1						
Subjective financial security	.29	.02	<.001	.34	.02	<.001
Model 4 – H2						
Social connectedness	.44	.02	<.001	.21	.02	<.001

Notes.: Each Model added variables to those included in the previous models. E.g., Model 3 added subjective financial security to the variables included in Models 1 and 2. β, *SE* and *p* are reported for the variable at the specific Model in which it was first entered

*DV* Dependent variable

H1: Hypothesis 1

H2: Hypothesis 2

**Fig. 1:**
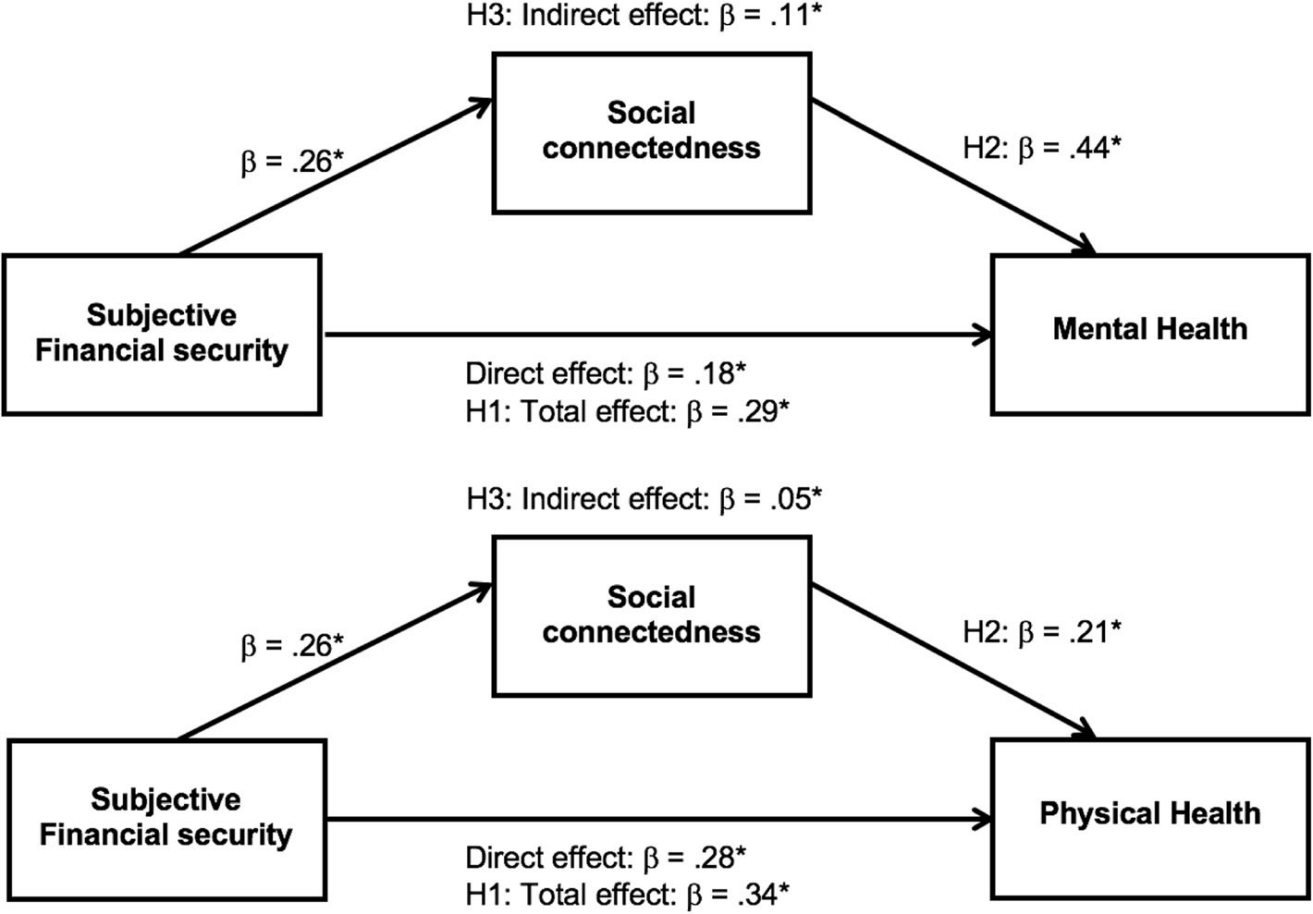
Population-weighted cross-sectional analysis: Financial security protects health, in part, because it enables social connectedness. *Notes. N* = 3109 retirees. These analyses include the covariates of gender, age, and education. Mixed effects mediation model includes random intercepts for households. *p < .001. H1: Hypothesis 1. H2: Hypothesis 2. H3: Hypothesis 3

Confirming H3, the mediation model found a significant indirect effect of subjective financial security on mental health via social connectedness, IE: .11, *p* < .001; CI: .10, .13. Approximately 39% of the relationship between subjective financial security and mental health was attributable to social connectedness. The mediation model is presented in Fig. 1a.

#### Physical health

The cross-sectional analysis was repeated for physical health as the dependent variable (see Table 2). Model 1 included random intercepts for household (accounting for 32% of the variance) and postcode (accounting for 3% of the variance). Model 2 included the covariates, and found people who were female and/or better educated were likely to experience better physical health. Confirming H1, Model 3 showed that subjective financial security significantly predicted physical health, β = .34, *p* < .001; CI: .30, .37. Confirming H2, Model 4 revealed that social connectedness also significantly predicted physical health, β = .21, *p* < .001; CI: .18, .25. Log-likelihood ratio tests between each subsequent model (1–4) were all significant χ^2^(1–4) = 39.98–318.11, *p*s < .001, indicating that model fit was improved with each additional predictor. Supporting H3, the mediation model confirmed a significant indirect effect of subjective financial security on physical health via social connectedness, IE: .05, *p* < .001; CI: .04, .07. Approximately 16% of the relationship between subjective financial security and physical health was attributable to social connectedness. The mediation model is presented in Fig. 1b.

### Longitudinal analyses

#### Mental health

Model 1 showed that random intercepts for household and postcode accounted for 57 and 4% of the variance in mental health, respectively. Model 2 found that none of the covariates were significant, perhaps in part because the pre-retirement measures of mental health (β = .56, *p* < .001; CI: .47, .64) and social connectedness (β = .22, *p* < .001; CI: .14, .30) accounted for much of the variance. Pre-retirement subjective financial security was not a significant predictor, β = .03, *p* = .405; CI: −.04, .11. Confirming H1, Model 3 showed that post-retirement subjective financial security significantly predicted better mental health in the retirement transition, β = .19, *p* < .001; CI: 09, .29. Confirming H2, Model 4 added post-retirement social connectedness, which significantly predicted better mental health in the retirement transition, β = .24, *p* < .001; CI: .14, .34. Log-likelihood ratio tests between each subsequent model (1–4) were all significant χ^2^(1–6) = 15.03–274.44, *p*s < .001, indicating that model fit was improved with each additional predictor. Full details of the longitudinal mixed effects models are provided in Table 3.

**Table 3:** Longitudinal linear mixed models assessing H1 and H2

	Longitudinal sample transitioning to retirement (*N* = 404)					
	DV: Mental health			DV: Physical health		
Model 1 – Random intercepts for household and postcode						
Model 2 - Covariates	β	*SE*	*p*	β	*SE*	*p*
Gender	−.06	.07	.417	−.03	.07	.639
Age	.00	.004	.980	.00	.004	.917
Education	.004	.01	.793	.02	.01	.206
Pre-retirement subjective financial security	.03	.05	.405	.10	.04	.004
Pre-retirement social connectedness	.22	.04	<.001	.10	.04	.005
Pre-retirement measure of health DV	.56	.04	<.001	.68	.04	<.001
Model 3 – H1						
Subjective financial security	.19	.05	<.001	.16	.05	<.001
Model 4 – H2						
Social connectedness	.24	.05	<.001	.11	.05	.016

Notes.

Each Model added variables to those included in the previous models. E.g., Model 3 added subjective financial security to the variables included in Models 1 and 2. β, *SE* and *p* are reported for the variable at the specific Model in which it was first entered. *DV* Dependent variable

H1: Hypothesis 1

H2: Hypothesis 2

**Fig. 2:**
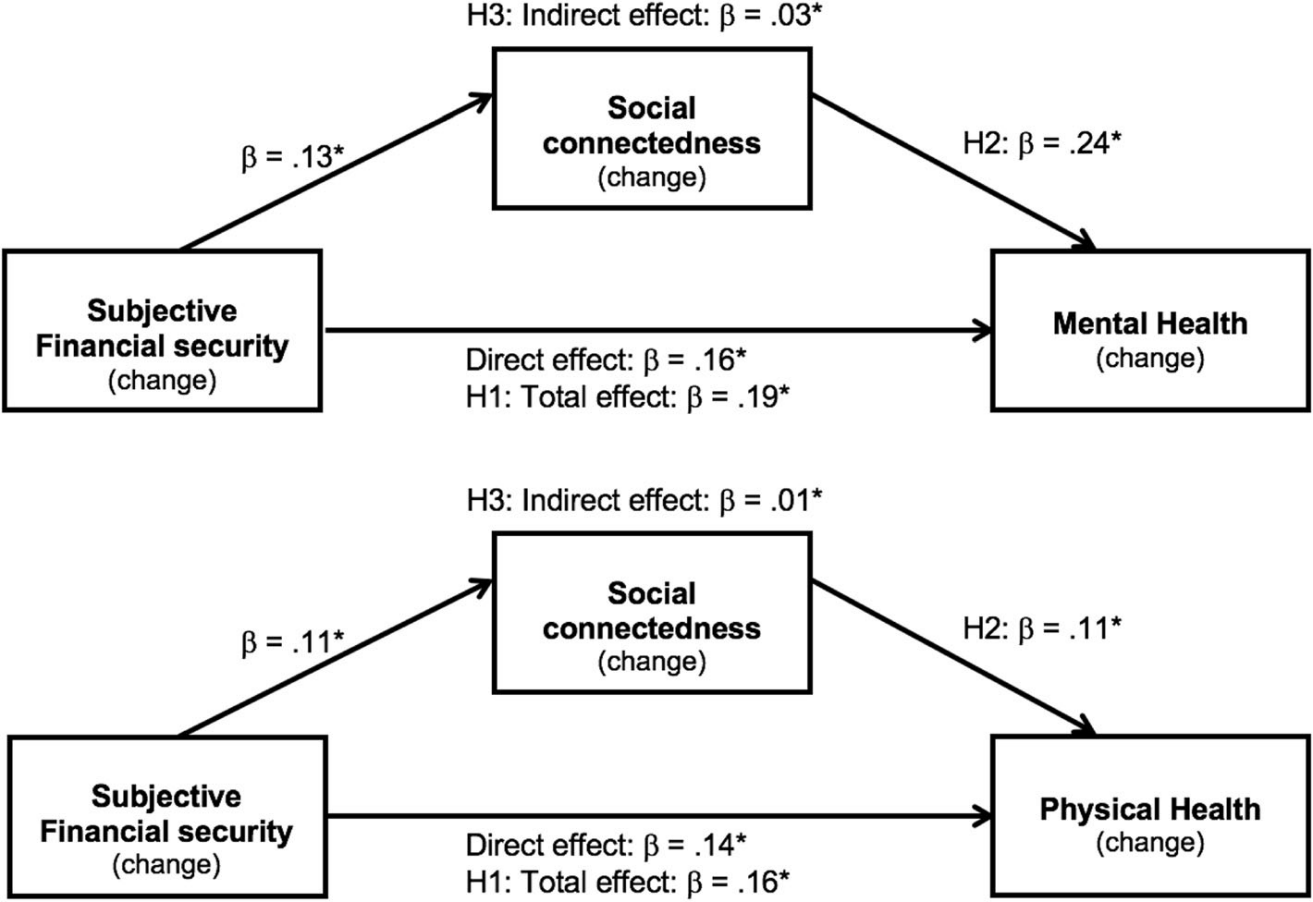
Longitudinal analysis: Financial security protects health, in part, because it enables social connectedness. *Notes. N* = 404 people transitioning to retirement. These analyses include the covariates of gender, age, and education, as well as pre-retirement measures of the three focal variables. Mixed effects mediation model includes random intercepts for households. *p < .001. H1: Hypothesis 1. H2: Hypothesis 2. H3: Hypothesis 3

To assess H3, a mediation analysis was conducted (see Fig. 2a). This model controlled for all three variables of interest at the pre-retirement timepoint, providing a conservative estimate of the proportion of variance that can be attributed to *change* in subjective financial security and social connectedness. This analysis confirmed a significant indirect effect of post-retirement subjective financial security via post-retirement social connectedness, IE: .03; *p* = .004; CI: .01, .06. Approximately 16% of the relationship between change in subjective financial security and change in mental health was attributable to change in social connectedness.

#### Physical health

Finally, the longitudinal analysis was repeated for physical health as the dependent variable (see Table 3). Model 1 included random intercepts for household (accounting for 52% of the variance) and postcode (accounting for 13% of the variance). Model 2 found that none of the covariates were significant, but that pre-retirement measures of physical health (β = .68, *p* < .001; CI: .61, .75), social connectedness (β = .10, *p =* .004; CI: .03, .17) and subjective financial security (β = .10, *p* = .003; CI: .03, .17) were each significant predictors. Confirming H1, Model 3 showed that post-retirement subjective financial security significantly improved the model, β = .16, *p* < .001; CI: .07, .25. Confirming H2, Model 4 revealed that social connectedness also significantly predicted physical health, β = .11, *p* = .016; CI: .02, .20. Log-likelihood ratio tests between each subsequent model (1–4) were all significant χ^2^(1–6) = 5.93–346.54, *p*s < .015, indicating model fit was improved with each additional predictor. Confirming H3, the mediation model indicated a significant indirect effect of subjective financial security on physical health via social connectedness, IE: .01; *p* = .046; CI: .004, .03. Approximately 8% of the relationship between subjective financial security and physical health was attributable to social connectedness (see Fig. 2b).

### Robustness checks

To verify the robustness of the findings, all cross-sectional and longitudinal analyses were repeated with the following changes (in all combinations):

1. using household income as the indicator of financial security, and;
2. without covariates of age, gender, and level of education.

These analyses supported H2 in all cases. H1 and H3 were supported in 12 of the 16 models run. Specifically, household income did not predict health (mental or physical) longitudinally, and so H1 and H3 were not supported for these analyses. These additional models are summarised in Additional file 1: Table S1. When averaged across the 16 models, the effect of social connectedness on health was 4.19 times stronger than the effect of financial security (based on the average difference between the Model 4 beta weights).

## Discussion

The present research used nationally representative data from Australian retirees to examine relationships between financial security, social connectedness, and health both cross-sectionally and longitudinally. Across multiple data treatments and samples, analyses revealed consistent and robust relationships among these variables showing that financial security predicted greater social connectedness, which in turn supported better health. This accords with two findings from previous research: first, that social connectedness is a critical protective factor for health [19], and, second, that financial security is important for retiree health and adjustment [3].

However, these findings also go beyond this prior work by providing new insight into why it is that financial security is important for retirees: because of its capacity to increase opportunities for social connection. Importantly, though, in showing that social connectedness was the means through which finances support health, the findings also suggest that investment in social planning to support connectedness might further enhance retiree outcomes. Underlining the importance of this point, social connectedness emerged as a more powerful and consistent predictor of health than financial security. Indeed, when their effect sizes are directly compared, social connectedness was on average four times stronger than financial security in predicting health. Accordingly, there would appear to be value in utilising social intervention in preventative ways, to develop and protect connectedness in critical periods of life transition such as in the lead up to and early stages of retirement [11]. Although research into social interventions is in its infancy, there is growing evidence that those programs which focus on building group-based ties and participation hold particular promise [42, 43].

As the above implications suggest, financial security alone is not the only means through which health is protected in retirement, and nor are financial resources the only means through which to increase social connectedness. This is particularly important among people with fewer financial resources — for whom investment in social planning and community engagement become paramount to support health and well-being in the retirement transition. Here it would appear that investment in social, alongside financial, services is critical and that government and corporate sectors are well placed to facilitate this. In much the same way that they take responsibility for providing information and education on building one’s retirement nest egg, these institutions are also in a position to inform and educate people about the benefits of social connectedness in supporting health in retirement.

### Strengths and limitations

A strength of the current analysis was the well-powered, high quality dataset which allowed us to approach the analyses in a number of different ways. The findings were robust to these different treatments, with the hypotheses (particularly regarding the centrality of social connectedness) supported for both physical and mental health, for both subjective and objective indicators of financial security, and both cross-sectionally and longitudinally. In addition, we were able to utilise multi-level modelling, and included both population weighting and demographic covariates. This increases our confidence that the findings are robust and unlikely to be attributable to confounds, are generalizable across retirees (at least within Australia), and are not specific to any one particular conceptualisation of financial security or health. However, it is nevertheless the case that only an experimental investigation can provide evidence of causality. In addition, a disadvantage of utilising existing datasets is that our analyses were limited by the variables available. These did not include, for instance, state-of-the-art measures of social connectedness, particularly social identification (see [32] for a review of measures), which has been shown to be a key construct in accounting for people’s health and well-being across a range of conditions and contexts [44].

## Conclusions

The present findings show that financial security offers retirees more than simply material comfort. In particular, they provide further evidence for a key conclusion from research on the social determinants of health: namely that the value of prosperity is not only monetary, but also derives from the less tangible resources that people need to live a good life. In the specific case of retirees that we have considered here, it appears that a decline in financial security brings with it risks of social isolation, and that this in turn is a major reason for subsequent risk of health decline. As a corollary, though, our research also suggests that a key benefit of achieving financial security in retirement is that this affords access to the socially curative benefits of an active social life. As others have observed, money may not buy you love, but it puts you in a much better bargaining position.

## Supplementary information

**Additional file 1: Table S1.** Sensitivity analyses using objective household income as the operationalisation of financial security.

## Data Availability

The data that support the findings of this study are available from the Melbourne Institute. Restrictions apply to accessing these data, which were used under license for the current study, and so are not publicly available. Data are however available from the authors upon reasonable request and with permission of Melbourne Institute.

## Abbreviations

CI: 95% confidence interval
DV: Dependent variable
H1: Hypothesis 1
H2: Hypothesis 2
H3: Hypothesis 3
HILDA: Housing, Income and Labour Dynamics in Australia survey
SES: Socioeconomic status
