# Supplementary material for: Friendships that money can buy: financial security protects health in retirement by enabling social connectedness
Source: BMC Geriatr. 2019 Nov 21;19:319. doi: 10.1186/s12877-019-1281-1 (PMC6868702; doi:10.1186/s12877-019-1281-1)
Supplement: Supplementary file 1 — Additional file 1: Table S1. Sensitivity analyses using objective household income as the operationalisation of financial security. [file 12877_2019_1281_MOESM1_ESM.docx]

**Additional file 1**

Table S1.

*Sensitivity analyses using objective household income as the operationalisation of financial security.*

|  | DV: Mental health | DV: Physical health |
| --- | --- | --- |
| Cross-sectional sample  (population weighted) | H1: β = .10; *p* < .001  H2: β = .48; *p* < .001  H3: β = .08; *p* < .001 | H1: β = .16; *p* < .001  H2: β = .27; *p* < .001  H3: β = .04; *p* < .001 |
| Longitudinal sample  (including pre-retirement measures of the three focal variables as covariates) | H1: β = .06; *p* = .248  H2: β = .25; *p* < .001  H3: β < .01; *p* = .91 | H1: β = .02; *p* = .735  H2: β = .15; *p* = .002  H3: β < .01; *p* = .77 |

*Notes.*

These analyses include covariates of gender, age, and education. These results, and those reported in the manuscript, were also replicated if covariates were excluded.

H1: Retirees’ financial security will predict their health.

H2: Retirees’ social connectedness will predict their health, after controlling for financial security.

H3: There will be an indirect effect of financial security on retirees’ health via social connectedness.
